# Variable Protease-Sensitive Prionopathy Transmission to Bank Voles

**DOI:** 10.3201/eid2501.180807

**Published:** 2019-01

**Authors:** Romolo Nonno, Silvio Notari, Michele Angelo Di Bari, Ignazio Cali, Laura Pirisinu, Claudia d’Agostino, Laura Cracco, Diane Kofskey, Ilaria Vanni, Jody Lavrich, Piero Parchi, Umberto Agrimi, Pierluigi Gambetti

**Affiliations:** Istituto Superiore di Sanità, Rome, Italy (R. Nonno, M.A. Di Bari, L. Pirisinu, C. d’Agostino, I. Vanni, U. Agrimi);; Case Western Reserve University, Cleveland, Ohio, USA (S. Notari, I. Cali, L. Cracco, D. Kofskey, J. Lavrich, P. Gambetti);; University of Bologna, Bologna, Italy (P. Parchi);; Istituto di Ricovero e Cura a Carattere Scientifico, Bologna (P. Parchi)

**Keywords:** Variable protease-sensitive prionopathy, VPSPr, prion disease, transmissibility, phenotype, bank voles, sporadic, Creutzfeldt-Jakob, Gerstmann-Sträussler-Scheinker, 109M, 109I, type 1, type 2, minor PrP components, lesion profile, 7 kDa, atypical glycoform, strain, substrain, bank voles, prions

## Abstract

Variably protease-sensitive prionopathy (VPSPr), a recently described human sporadic prion disease, features a protease-resistant, disease-related prion protein (resPrP^D^) displaying 5 fragments reminiscent of Gerstmann-Sträussler-Scheinker disease. Experimental VPSPr transmission to human PrP–expressing transgenic mice, although replication of the VPSPr resPrP^D^ profile succeeded, has been incomplete because of second passage failure. We bioassayed VPSPr in bank voles, which are susceptible to human prion strains. Transmission was complete; first-passage attack rates were 5%–35%, and second-passage rates reached 100% and survival times were 50% shorter. We observed 3 distinct phenotypes and resPrP^D^ profiles; 2 imitated sporadic Creutzfeldt-Jakob disease resPrP^D^, and 1 resembled Gerstmann-Sträussler-Scheinker disease resPrPD. The first 2 phenotypes may be related to the presence of minor PrP^D^ components in VPSPr. Full VPSPr transmission confirms permissiveness of bank voles to human prions and suggests that bank vole PrP may efficiently reveal an underrepresented native strain but does not replicate the complex VPSPr PrP^D^ profile.

Sporadic prion diseases are classified according to phenotype as well as the pairing of the prion protein (PrP) genotype at the methionine (M)/valine (V) polymorphic codon 129 and the conformational characteristics of the abnormal or disease-associated PrP (PrP^D^). These characteristics include electrophoretic mobility and the ratio of the PrP^D^ fragments that are resistant to proteinase K (PK) digestion (Appendix Table 1) (*1*). According to these criteria, the 3 major types of sporadic prion disease are sporadic Creutzfeldt-Jakob disease (sCJD), sporadic fatal insomnia, and variably protease-sensitive prionopathy (VPSPr) (*2*–*5*).

VPSPr was first reported in 2008 and further defined in 2010 (*6*–*8*) as a sporadic prion disease distinct from sCJD. Since then, 37 cases have been reported, consistent with a prevalence rate of 1%–2% for all sporadic prion diseases (*8*). Similar to sCJD, VPSPr targets all 3 PrP genotypes. However, the prevalence of the 3 genotypes at codon 129 (MM, MV, and VV) greatly differs, indeed is almost inverted, in the 2 diseases: homozygosity VV is the most common (65%) genotype in VPSPr and the least common (16%) in sCJD (*2*,*9*). Furthermore, at variance with sCJD, in which the 129 genotype is a determinant of disease phenotype and PrP^D^ characteristics, the 129 genotype influence on phenotype, although present, is subtle (*3*,*7*,*8*). These differences point to a distinct role of the 129 genotype as a risk factor and imply that the etiologic-pathogenetic mechanisms of the 2 diseases differ.

Although the histopathology of VPSPr is distinct (e.g., spongiform degeneration, frequent presence of PrP microplaques, and a recognizable PrP^D^ immunostaining pattern), the hallmarks of VPSPr are the characteristics of its PrP^D^. In contrast to virtually all other sporadic human prion diseases, in which PK-resistant PrP^D^ (resPrP^D^) electrophoretically separates into 3 major bands, VPSPr resPrP^D^ characteristically separates into 5 bands. Furthermore, although the 3 bands of resPrP^D^ are all cleaved by PK exclusively at the N terminus and separate according to the presence of 2, 1, or 0 sugar moieties, VPSPr resPrP^D^ bands include only the monoglycosylated and unglycosylated forms, which are cleaved either only at the N terminus or at both the N- and C-termini. Thus, the C-terminus–truncated resPrP^D^ lacks the GPI (glycosylphosphatidylinositol) anchor. Additional variances concerning immunoreactivity characteristics, ratios of PK-resistant and PK-sensitive PrP^D^ species, and conformational properties including aggregate size, have also been observed (*6*–*8*). These distinctive properties point to VPSPr PrP^D^ as a prion strain different from those of other sporadic prion diseases. However, the VPSPr prion shares the multiplicity of the resPrP^D^ electrophoretic bands with prions from a subset of inherited prion diseases referred to as Gerstmann-Sträussler-Scheinker disease (GSS), prompting the suggestion that VPSPr is the sporadic form of GSS (*7*,*10*). Furthermore, the presence of small amounts of sCJD-like 3-band resPrP^D^ has also been signaled in VPSPr (*6*,*11*,*12*).

Disease transmission to receptive hosts is a valuable way to further define the characteristics of strains associated with prion diseases. VPSPr has been experimentally transmitted to 3 lines of transgenic mice expressing normal PrP or cellular human PrP (PrP^C^), harboring residue M, V, or MV at residue 129 (*13*,*14*). Data in all experiments were essentially similar. Inoculated mice remained asymptomatic, but half showed focal PrP^D^ plaques with minimal spongiform degeneration, and PrP^D^ mimicking the electrophoretic profile of the native PrP^D^ on immunoblot was demonstrated in about one third of the inoculated mice. No transmission was observed at second passage.

The bank vole, a small rodent resembling the mouse with which it shares the entire sequence of normal PrP or PrP^C^ except for 8 aa, but whose sequence differs from human PrPC by 15 aa, has recently emerged as a particularly permissive host. Bank voles and transgenic mice expressing bank vole PrP^C^ have been successfully infected after challenge with human and animal prion diseases that are hard to transmit even to recipients expressing homologous PrP^C^ (*15*–*18*).

We studied transmission of VPSPr from patients with MM, MV, and VV codon 129 genotypes to bank voles harboring either the PrP genotype 109M (bv109M) or 109I (bv109I). Although the attack rate was generally low at first passage, it consistently raised to 100% at second passage, when survival times also decreased on average by >50%. We identified 3 PrP^D^ isoforms with the characteristics of distinct strains in the affected bank voles.

## Materials and Methods

The inocula used in the first passage were brain homogenates from 7 persons with a definitive diagnosis of VPSPr: 2 with genotypes 129MM, 3 with 129MV, and 2 with 129VV. Homogenate was inoculated into the cerebrum of 205 bank voles according to previously described procedures (*16*). The bank vole brains were processed for histopathology, immunohistochemistry, lesion profiles, and paraffin-embedded tissue (PET) blots according to previously reported procedures (*15*). Western blot was performed according to Notari et al. (*19*). The insoluble fraction was prepared according to previously described procedures (*20*). Preparation of monoclonal antibodies is described in the Appendix. Statistical significance was determined by 1-way analysis of variance, followed by the Tukey multiple comparison test.

## Results

### Transmission Characteristics

At first passage, attack rates of VPSPr were 35% (29/82) in bv109I and 5% (3/59) in bv109M (Table 1; Appendix Table 2). The 2 bank vole genotypes diverged as to disease transmission in 2 ways. First, all VPSPr 129 genotypes were transmitted to bv109I, but bv109M were not susceptible to VPSPr-VV. Second, bv109I propagated 3 distinct histopathologic phenotypes and matching PrP^D^ types (hereafter identified as T1, T2, and T3), but bv109M replicated the T1 phenotype exclusively. A more detailed analysis in bv109I, although limited by the low number of animals in each subset, suggested a lower attack rate for VPSPr-VV, the most common form of human VPSPr, compared with the MM and MV genotypes and a prevalence for T3 that was 11% higher than that for T1 and 106% higher than that for T2 (Table 2). Overall survival times were 575 days postinoculation (dpi) for bv109I and 411 dpi for bv109M. However, when we considered only the bank voles associated with the T1 phenotype, because bv109M were exclusively associated with T1, the dpi difference became smaller: 490 dpi for bv109I and 411 dpi for bv109M (Tables 1, 2). As for survival times related to T1–T3 phenotypes and VPSPr genotypes, the survival times for T2 were nearly twice those for T1 and T3 (Table 2).

**Table 1:** VPSPr transmission to bank voles*

Inoculum	Bv109I									Bv109M						
	1st passage				2nd passage					1st passage				2nd passage		
	PrP^D^ type	Attack rate	Survival time, dpi ± SD		PrP^D^ type	Attack rate	Survival time, dpi ± SD	Survival reduction, %		PrP^D^ type	Attack rate	DPI		PrP^D^ type	Attack rate	Survival time, dpi ± SD
VPSPr-MM, n = 2	T1	1/20†	901		NA	NA	NA	NA		T1	1/15	356		T1	11/11	148 ± 12
	T2	1/20	839		NA	NA	NA	NA								
	T3	5/20	413 ± 102		T3	9/9	247 ± 35‡	40								
VPSPr-MV, n = 3	T1	13/44†	458 ± 137		T1	10/10	195 ± 9§	57		T1	2/30	290, 588		T1	11/11	142 ± 11
	T2	6/44	872 ± 110¶		T2	14/14	338 ± 100#	61								
	T3	1/44	554		NA	NA	NA	NA								
VPSPr-VV, n = 2	T3	2/18	596, 535		T3	9/9	255 ± 24**	55		NA	0/14	NA		NA	NA	NA

*bv, bank voles; dpi, days postinoculation; NA, not available; neg, negative; PRP^D^, disease-associated prion protein; VPSPr, variably protease-sensitive prionopathy. †Total no. bank voles inoculated with either VPSPr-MM or VPSPr-MV; statistics of incubation periods at 1st vs. 2nd passages. ‡p<0.05. §p<0.0001. ¶Incubation periods of T2 vs. T1 p<0.0001. #p<0.0001. **p<0.001.

**Table 2:** Itemized VPSPr transmission features in bv109I at first passage, by phenotype*

Genotype	T1			T2			T3		Attack rate for all phenotypes, %
Prevalence, %	Survival time, dpi ± SD	Prevalence, %	Survival time, dpi ± SD	Prevalence, %	Survival time, dpi ± SD
MM	5	901		5	839		25	413 ± 102†	35
MV	29.5	458 ± 137†		13.6	872 ± 110†		2.3	554	45.5
VV	0	NA		0	NA		11.1	596, 535	11.1
All affected genotypes	11.5‡	490 ± 177†		6.2‡	867 ± 101†		12.8‡	469 ± 110†	30.5‡

*bv, bank voles; dpi, days postinoculation; VPSPr, variably protease-sensitive prionopathy. †Weighted average ± SD. ‡Unweighted average.

Second passage in bv109I was invariably characterized by a 100% attack rate, a 40%–61% decrease in survival times, and conservation of the original phenotype (Table 1). A similar trend was observed for bv109M.

### Histopathology and Immunohistochemistry

**Figure 1:**
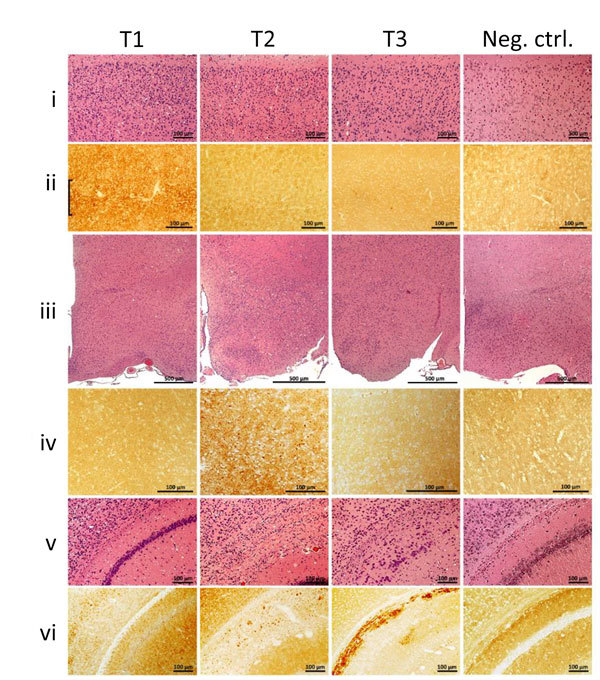
Histopathology and prion protein (PrP) immunohistochemistry images of brain regions from variably protease-sensitive prionopathy (VPSPr)–inoculated bank voles 109I harboring the histopathologic phenotypes T1, T2, or T3. For T1 bank voles, the cerebral neocortex (i, ii) shows moderate spongiform degeneration with substantial PrP immunostaining often displaying a laminar enhancement (bracket), but spongiform degeneration and PrP immunostaining were minimal or lacking in T2 and T3 bank voles (i, ii, T2, T3). By contrast, spongiform degeneration and immunostaining characterized by granular aggregates were selectively prominent in the hypothalamus of T2 but not in T1 and T3 (iii, iv) bank voles. Distinct lesions also occurred in a region comprising dorsal hippocampus and overlying hemispheric white matter (v, vi) where T1 shows hippocampal spongiform degeneration with fine disease-related prion protein (PrP^D^) deposition; T2 shows granular or small plaque-like PrP^D^ deposition affecting primarily the alveus; and T3 shows an intense and compact PrP^D^ deposition affecting stratum oriens, alveus, and white matter of the corpus callosum. The negative control (Neg Ctrl) column (i–vi) shows tissue from inoculated bank voles that were negative by Western blot. Neither spongiform degeneration nor positive PrP immunostaining was observed in these controls. All panels T1–T3 are from second passage; antibody SAF84. Scale bars for row iii indicate 500 μm; all others indicate 100 μm. This figure is also available online at https://wwwnc.cdc.gov/EID/article/25/1/18-0807-F1.htm.

**Figure 2:**
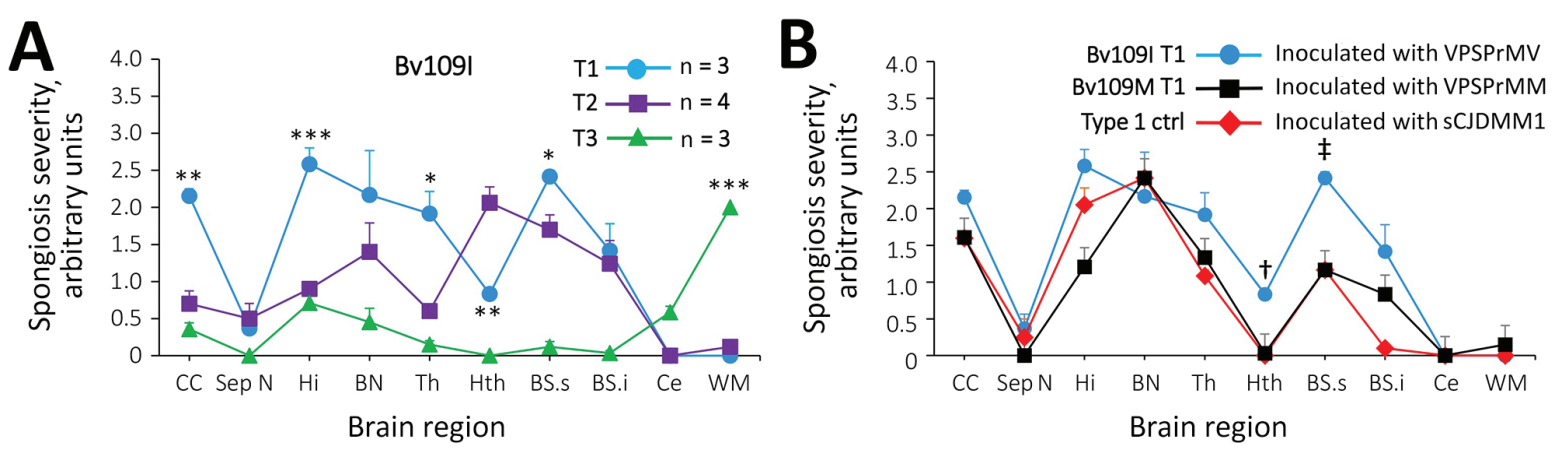
Profiles of topographic distribution and severity of spongiform degeneration in the brains of bank voles harboring T1–T3 phenotypes after inoculation with brain homogenate from variably protease sensitive prionopathy (VPSPr) and control bank voles inoculated with sCJD. A) Spongiform degeneration characterized both T1 and T2 phenotypes but displayed significantly divergent distributions in 5 of the 10 anatomic locations examined; spongiform degeneration affected primarily the cerebral cortex in T1 and the hypothalamus and brain stem in T2; no difference in vacuolar mean diameter was observed between T1 and T2. Spongiform degeneration scores associated with the T3 phenotype were minimal or absent in most locations except for the white matter, especially in the corpus callosum, which was virtually unaffected in T1 and T2. *p<0.5; **p<0.006; ***p<0.0001 of T1 versus T2 and T3 WM versus T1 and T2; inocula: T1 and T2 VPSPr-129MV, T3 VPSPr-129MM; vacuoles measured (n = ≈2,000) in T1 and T2 combined. B) Comparative study of T1 profiles generated in bv109M and bv109I revealed an overall more severe spongiform degeneration in bv109I but no significant difference in distribution (†, p<0.001, ‡, p<0.003; N = 3 Bv109I and Bv109M). The T1 spongiform degeneration profile generated by bv109M after inoculation with VPSPr-129MM reproduced the profile generated with sCJDMM1 extracts used as control for human type 1 (bv109M N = 3 for each profile). Similar results were obtained when comparing the T1 profile of bv109I inoculated with VPSPr-129MV and profiles of bv109I inoculated with sCJDMV1 (data not shown). BN, basal nuclei; BSs and BSi, brainstem superior and inferior; bv, bank vole; CC, cerebral cortex; Ce, cerebellum; ctrl, control; Hi, hippocampus; Hth, hypothalamus; sCJD, sporadic Creutzfeldt-Jakob disease; Sept.N, septal nuclei; Th, thalamus; WM, white matter.

**Figure 3:**
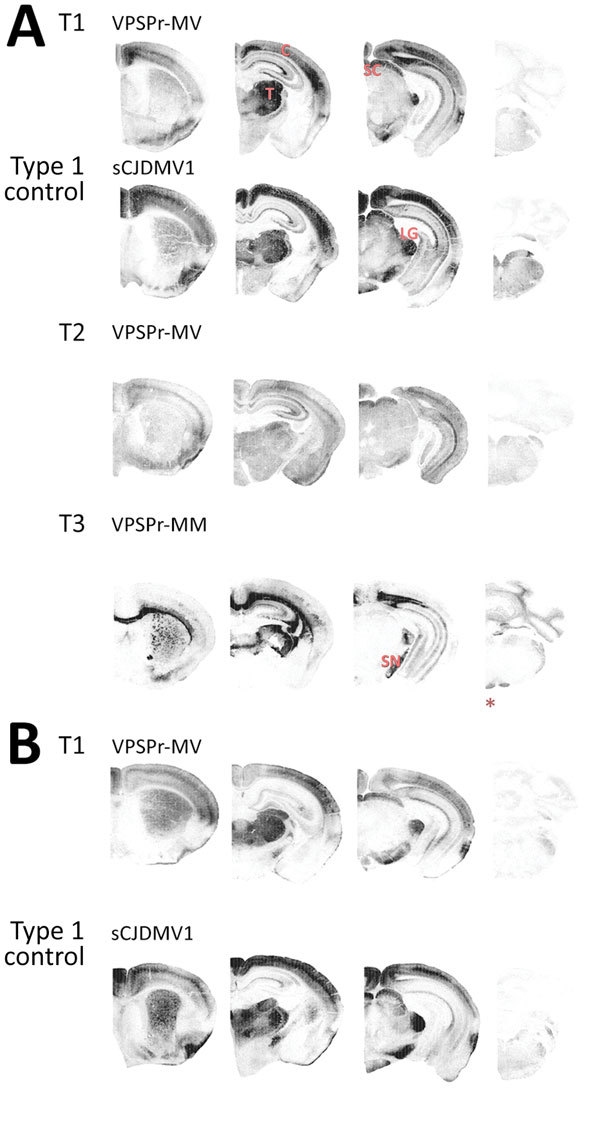
Representative paraffin-embedded tissue (PET) blots of protease-resistant, disease-related prion protein (resPrP^D^) distribution in phenotypes T1–T3 and controls. A) For T1, PrP^D^ predominated in cerebral cortex (C), thalamus (T), superior colliculus (SC), lateral geniculate nucleus (LG), and substantia nigra (SN). A similar PrP^D^ distribution was observed with transmission of sCJDMV1 used as type control. T2 showed a more uniform distribution in cerebral cortex and subcortical nuclei of an apparently lesser amount of PrP^D^; T3 appeared to preferentially affect the hemispheric white matter and other subcortical regions such as the alveus, the corpus callosum, the anterior commissure, and fascicles surrounding thalamus as well as other white matter formations such as fimbria, brachium of superior colliculus, medial lemniscus, and cerebral peduncles. Small amounts of PrP^D^ were also observed in cerebellar and medullary white matter (asterisk [*]). B) PrP^D^ T1 distribution resembled that of bank voles (bv) 109I after transmission of the same VPSPr-MV brain homogenate (compare with T1 in A). A similar distribution was also observed after inoculation with sCJDMV1. Left to right: coronal sections of telencephalon midlevel caudate nucleus; diencephalon midlevel thalamus; midbrain; and hindbrain-level medulla and cerebellum. sCJD, sporadic Creutzfeldt-Jakob disease; VPSPr, variably protease-sensitive prionopathy.

Phenotype T1 featured finely vacuolated spongiform degeneration often involving the entire thickness of the neocortex, including the molecular layer but occasionally also showing a laminar distribution (Figure 1). On second passage, the spongiform degeneration appeared to be more widespread, also affecting the hippocampus and subcortical structures such as basal nuclei, thalamus, and superior colliculi but not the cerebellum. PrP immunohistochemistry demonstrated punctate deposits often co-distributed with spongiform degeneration (Figure 1, column T1, row ii). At second passage, T1 features did not differ significantly between bv109M and bv109I. Furthermore, T1 also resembled the histopathologic phenotype shown by bv109M and bv109I after inoculation with sCJDMM1 or sCJDMV1, respectively (Figures 2, 3; Appendix Figure 1)

In phenotype T2, spongiform degeneration affected predominantly subcortical structures over neocortical regions, especially the hypothalamus with the apparent exclusion of the mammillary bodies (Figure 1, column T2, row iii). PrP immunohistochemistry showed granular deposits occasionally resembling miniature plaquelike formations rather than the punctate deposits of the T1 phenotype (Figure 1, column T2, row iv).

Phenotype T3 was characterized by the paucity of spongiform degeneration in the cerebral neocortex and subcortical gray matter structures; spongiform degeneration was often prominent in the regions of the hemispheric white matter lying above the hippocampus and in the corpus callosum, where parenchyma was occasionally disorganized with glial reaction. PrP immunostaining was mostly limited to those regions where it often aggregated in confluent plaque-like deposits but not well-formed plaques (Figure 1, column T3, row v, and column Tc, row vi). No remarkable differences were detected between first and second passages. Overall, the T3 histopathologic phenotype resembled that shown by bv109I after inoculation with brain homogenates from some GSS subtypes (*16*).

It is noteworthy that the T1–T3 phenotypes were never observed to coexist in 1 animal, although distinct phenotypes were often observed in bank voles receiving the same inoculum. Although all 3 phenotypes occurred after inoculation with VPSPr-MM or -MV, the sole phenotype associated with VPSPr-VV inoculation was T3 (Table 1).

### Lesion Profiles and PET Blots

Profiles of spongiform degeneration as a function of lesion severity and brain anatomic location confirmed the distinctive characteristics of the T1–T3 phenotypes (Figure 2, panel A; Appendix Figure 1). The T1 spongiform degeneration profile in bv109I did not differ significantly from that of bv109M; both mirrored the profiles of bv109I inoculated with sCJDMV1 and bv109M inoculated with sCJDMM1 brain homogenate (Figure 2, panel B; Appendix Figure 1).

The PET blot patterns of brain PrP^D^ distribution were also quite distinct in the 3 phenotypes and, overall, reproduced the spongiform degeneration distribution (Figures 2, 3). In T1, PrP^D^ was well represented in selected regions including cerebral neocortex and hippocampus, basal nuclei, thalamus, superior colliculi, geniculate nuclei, and substantia nigra but not in the cerebellum and lower brain stem. No significant variations were detected between PrP^D^ distributions at first and second passages (data not shown). PrP^D^ distributions were also similar in bv109I and bv109M inoculated with classic sCJDMV1 and sCJDMM1 prions, respectively (Figure 3, panel B). In the T2 phenotype, PrP^D^ appeared to be present in moderate and uniform amounts in several anatomic regions such as neocortex and hippocampus, thalamus, and superior colliculi (Figure 3). The T3 phenotype was characterized by the striking presence of PrP^D^ in hippocampus and white matter structures (Figure 3).

### PrP^D^ Characterization

**Figure 4:**
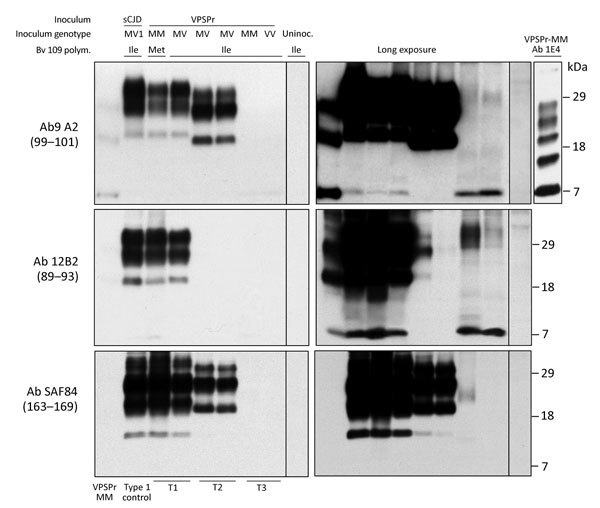
Immunoblot characteristics of protease-resistant, disease-related prion protein (resPrP^D^) distribution in phenotypes T1–T3 and controls. Regular and long exposures revealed the overall similarity of the 3-band profiles in T1 and T2, but resPrP^D^ profile, including glycoform representation, differed in the 2 phenotypes with all 3 monoclonal antibodies (Ab) used. T1 included a 7-kDa band, not detected in T2, similar to mobility and Ab immunoreactivity of the T3 7-kDa fragment. The T1 profile matched the profile generated in isogenic bank voles inoculated with sCJDMV1 used as human resPrP^D^ type 1 control (ctrl) (lane 2). The T3 profile, visible only after long film exposures, featured a 7-kDa band, but slower migrating bands with variable immunoreactivity were also visible. None of the T1–T3 profiles matched the original VPSPr profile (first lane) although the ≈7-kDa and both 23-kDa and 19-kDa bands were shared with T1 and T2, respectively (compare first with T1 and T2 lanes). The complexity of the native resPrP^D^ profile from VPSPr homogenate is demonstrated by probing with 1E4, a monoclonal Ab to human PrP highly reactive to VPSPr resPrP^D^ (top right panel) (*6*). Monoclonal Ab 12B2 (middle panels) with high affinity for human resPrP^D^ type 1 confirmed the type 1 characteristics of the resPrP^D^ associated with the T1 phenotype. The small amount of resPrP^D^ type 1 in 1 T2 bank vole probably represents incomplete proteinase K (PK) digestion (lane 5, right panel) (*19*). Monoclonal Ab SAF84 to the PrP C-terminus, unreactive to human PrP, further underlined the divergence in resPrP^D^ primary structure in T1 and T2 compared with T3. Aside from revealing an additional 13-kDa fragment, strongly detected in T1 and T2 and weakly in T3, SAF84 did not detect the 7-kDa fragment, supporting its internal origin (i.e., cleaved at both N- and C-termini). Uninoculated bank voles were negative for resPrP^D^. All samples were PK treated. sCJD, sporadic Creutzfeldt-Jakob disease; uninoc., not inoculated; polym., polymorphism; VPSPr, variably protease-sensitive prionopathy.

**Figure 5:**
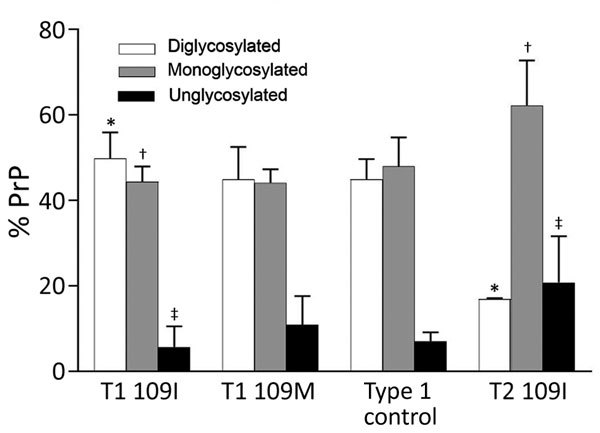
Glycoform ratio of protease-resistant, disease-related prion protein (resPrP^D^) in phenotypes T1 and T2. The ratio of resPrP^D^ associated with T1 (T1 109I) was 48% for diglycosylated, 44% for monoglycosylated, and 8% for unglycosylated conformers and significantly differed in each glycoform from the 17%, 63%, and 20% corresponding ratio of T2 (T2 109I). *p<0.0001; †p<0.005; ‡p<0.05. Glycoform ratios of T1 109I and T1 109M as well as that of type 1 control (from bank voles 109I inoculated with sporadic Creutzfeldt-Jakob disease MV1 and used as human type 1 controls) did not significantly differ from each other. Each bar represents mean ± SD of n = 4 for T1 109M, n = 6 for T1 109I, n = 6 for T2, and n = 2 for type 1 control.

Immunoblot analysis confirmed the presence of 3 distinct resPrP^D^ electrophoretic profiles that matched the 3 histopathologic phenotypes. When probed with antibodies 9A2 and 12B2, resPrP^D^ associated with the T1 phenotype populated 3 bands of ≈32, 26, and 21 kDa, representing the 3 resPrP^D^ glycoforms, and by a fragment of 7 kDa (Figure 4). An additional C-terminal fragment of ≈13 kDa, possibly homologous to the human C-terminal fragment 12/13 (*20*), was detected by the C-terminal antibody SAF84 (Figure 4). Glycoform ratios showed a comparable representation of the diglycosylated and monoglycosylated forms of resPrP^D^ (Figure 5; Appendix Figure 2). The electrophoretic profile and glycoform ratios of resPrP^D^ T1 conformer were indistinguishable from those of resPrP^D^ observed in bank voles inoculated with sCJDMM1 or sCJDMV1 prions, used as controls for human resPrP^D^ type 1 in bank voles (Figures 4, 5; Appendix Figure 2; data not shown).

The resPrP^D^ profile associated with the T2 phenotype showed 3 bands of ≈30, 24, and 19 kDa (i.e., all that had an ≈2-kDa faster electrophoretic mobility than the corresponding bands of resPrP^D^ T1) (Figure 4). The 7-kDa fragment was not detected in T2 (Figure 4). In contrast to T1, the T2 glycoform ratio was characterized by the unambiguous predominance of the monoglycosylated component (Figure 5). In summary, bank vole resPrP^D^ T2 differed from the T1 conformer by overall 2-kDa faster mobility, the absence of the 7-kDa fragment, and marked predominance of the monoglycoform. The striking feature of the resPrP^D^ associated with the T3 phenotype was the predominant presence of the 7-kDa fragment detected by 9A2 and 12B2 but not by SAF84, demonstrating its internal origin and the absence of glycosylation sites (Figure 4).

**Figure 6:**
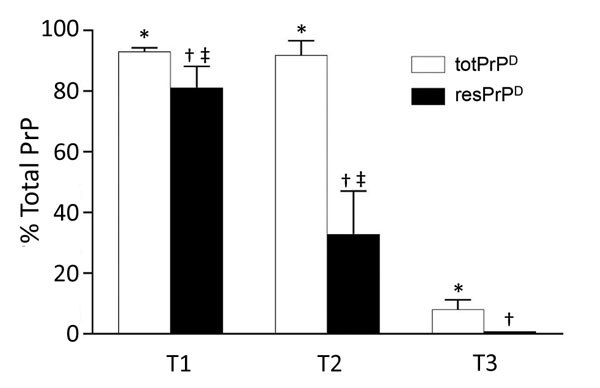
Relative quantities of totPrP^D^ and resPrP^D^ in T1–T3 phenotypes. totPrP^D^ accounted for 93.1% and resPrP^D^ for 81.3% of total PrP recovered from bank voles harboring the T1 phenotype. Corresponding percentages for T2 were 91.0% and 33.0%, and T3 totPrP^D^ and resPrP^D^ accounted only for 8.0% and 0.2% of total PrP and differed significantly from both T1 and T2 in each of the 2 components. ResPrP^D^ also differed significantly between T1 and T2 (each bar represents the mean ± SD of n = 3 T1, n = 3 T2, and n = 5 T3; all data are from bank voles 109I; antibody 9A2). bv, bank vole; resPrP^D^ protease-resistant, disease-related prion protein; totPrPD, comprising protease-sensitive PrP^D^ and resPrP^D^. *p<0.0001; †p<0.0001 vs. T1 and p<0.05 vs. T2; ‡p<0.01.

Additional divergent features emerged when amounts of totPrP^D^ (i.e., PK-sensitive plus resPrP^D^ fractions) were assessed as percentages of total PrP, comprising PrP^C^ and totPrP^D^ (Figure 6). A significantly larger component of totPrP^D^ was resPrP^D^ in T1 than in T2 (81% vs. 33%); totPrP^D^ fractions were similar (93% for T1, 91% for T2). T3 differed significantly: totPrP^D^ accounted for 8% and resPrP^D^ accounted for 0.2% of total PrP (Figure 6; Appendix Figure 3).

## Discussion

The permissiveness of bank vole PrP^C^ is well known (*15*,*16*,*18*,*21*–*27*); it is exemplified by the observation that, despite the mere 8-aa PrP^C^ divergence between bank voles and mice, a variety of human and animal prion diseases not transmissible to mice are infectious to bank voles and transgenic mice expressing bank vole PrP (*15*,*16*,*18*,*22*,*24*). Conversely, the 15-aa difference from the human PrP^C^ primary sequence does not impede the efficient transmission of a wealth of sporadic and inherited human prion diseases (*15*,*16*). This striking permissiveness has been attributed to the presence of several asparagine and glutamine residues in and around the β2–α2 loop that would result in a PrP^C^ conformation compatible with the conformations of a large number of PrP^D^ strains (*21*). Furthermore, the polymorphism at bank vole codon 109 adds further complexity to the interaction with exogenous strains (*18*,*28*).

We undertook systematic transmission of VPSPr brain homogenates to bv109M and bv109I after failure to consistently transmit VPSPr to humanized transgenic mice. Overall, transmission was favored by the 109I genotype, which propagated all 3 VPSPr 129 genotypes while bv109M failed to transmit VPSPr-VV. However, at first passage in bv109I, the mean attack rate (35%) was fairly low and the mean survival time (575 dpi) quite extended (Table 1). These conditions changed at second passage, when the attack rate became 100% in all transmission experiments and survival times decreased on average by 53% (Table 1). These findings point to the existence of a substantial barrier at first passage, which, judging from the 100% attack rate, is probably largely diminished or vanished at second passage. In view of the aforementioned easy transmissibility of other human prion diseases, the barrier appears to be conformational rather than caused by species-related variations in amino acid sequence of PrP^C^ (*15*,*16*); the barrier might be associated with the misfolding of VPSPr PrP^D^, which may be peculiar because after PK digestion it results in an array of highly heterogeneous fragments and apparently the failure to convert one of the glycoforms (*6*,*7*). Similarly, the clear effect of the genotype at codon 129 on the attack rate (which was 3–4 times lower for bank voles inoculated with VPSPr-VV prions compared with VPSPr-MM and -MV), along with the lack of transmission of VPSPr-VV to bv109M but not to bv109I, points to conformational differences between PrP^D^ species associated with the 129 genotypes in VPSPr (*16*). This notion is further supported by previous data showing higher PK sensitivity (*7*) and conformational stability of PrP^D^ (*29*) in VPSPr-VV compared with VPSPr-MM and -MV.

The comparative study of VPSPr bioassay in bank voles and humanized transgenic mice revealed substantial differences. VPSPr-challenged mice invariably remained asymptomatic, and all histologically positive mice failed to transmit at second passage. Furthermore, the VPSPr-MV subtype was never transmitted to mice 129M or 129V, and the general attack rate (assessed histopathologically) was low (54%); resPrP^D^ was demonstrated in only 34% of the challenged mice despite the 2–8 times normal levels of PrP expression for most mice (*13*). However, in contrast to bank voles, positive mice generated a resPrP^D^ conformer very similar to that of VPSPr for electrophoretic profile, glycosylation pattern, and antibody immunoreactivity, although it exhibited higher protease resistance.

Data from a previous study of transmission to humanized transgenic mice and bv109M of an sCJDMV variant with an atypical glycoform profile (CJD-MV^AG^) partially resembled ours (*17*). Challenged transgenic mice remained asymptomatic and negative at neuropathologic examination, but 22% of them reproduced the original resPrP^D^ electrophoretic profile and glycotype of the inoculum. In contrast to humanized transgenic mice, bank voles had full-blown disease develop featuring 3, although partially merging, histopathologic phenotypes along with 3 distinct resPrP^D^ conformers, none of which mimicked the profile and glycotype of the inoculum (*17*). Remarkably, the glycoform variation of sCJDMV^AG^ resembles that of VPSPr because both resPrP^D^ species lack the diglycosylated isoform, implicating this variation as one of the possible causes of bank vole failure to accurately replicate exogenous PrP^D^ (*17*).

Three subtypes of GSS (which VPSPr resembles in terms of the ladder-like electrophoretic profile and the sensitivity to PK of resPrP^D^) have also recently been transmitted to bank voles and 1 GSS subtype to humanized transgenic mice (*16*,*30*). Despite the well-known difficulty of transmitting GSS to rodents, bank voles challenged with 2 major GSS subtypes associated with PrP mutations A117V and F198S (GSS^A117V^, GSS^F198S^) showed no evidence of species or mutation barrier. Transmission was comparatively more difficult with the third GSS^P102L^ subtype, in which resPrP^D^ displays 2 sets of fragments: either the 8-kDa fragment associated with the 30–21 kDa glycoform triplet (*31*,*32*) or the 8-kDa fragment alone. After inoculation, the 2-fragment set was never replicated, and the ≈8-kDa fragment alone occasionally was inaccurately reproduced as a 7-kDa fragment (*16*,*28*). To date, only GSS^A117V^ has been transmitted to 2 lines of transgenic mice expressing human PrP^D^ harboring the A117V transition (*30*). Although transmission features diverged in the 2 lines, both seemed to reproduce the 7-kDa fragment that is the only strongly resPrP^D^ fragment in this disease.

Combined, these experiments indicate that PrP^C^ characteristics, and possibly other host factors (*25*), enable bank voles to be more permissive hosts (despite the species barrier) than transgenic mice expressing conspecific PrP^C^, confirming the empirical aspect of the species barrier. However, bank vole PrP^C^ can hardly reproduce faithfully complex features of human atypical prion isolates, a task that may require PrP^C^ from the same species.

A remarkable finding of this study is the occurrence of 3 well-defined histopathologic phenotypes (T1–T3), which displayed discrete PrP^D^ brain distribution and were linked to PrP^D^ conformers easily distinguishable by electrophoretic profile and glycosylation characteristics. The 3 phenotypes also differed by mean survival times at first and second passages. Remarkably, the T1–T3 phenotypes were often generated by the same inoculum but never co-occurred in the same bank vole. Combined, these features define the T1–T3 PrP^D^ conformers as distinct strains, raising the issue of their origin. Both histopathologic and resPrP^D^ electrophoretic characteristics of the T1 phenotype are essentially indistinguishable from those of bank voles inoculated with sCJDMV1. Data on transmission of sCJDMM2, available only for bv109M, show that the electrophoretic profile of the newly formed resPrP^D^ matches the T2 resPrP^D^ of this study (*15*). Although the T1 and T2 representations of totPrP^D^ and resPrP^D^ are not known in bank voles inoculated with sCJDMM1 and sCJDMM2 prions, the values we observed after VPSPr inoculation are comparable to those reported for the original sCJD, in which totPrP^D^ and resPrP^D^ reportedly accounted for 53.5% and 48.2% of total PrP in sCJDMM1 (*6*; L. Cracco et al., unpub. data). Therefore, transmission to bank voles suggests that VPSPr PrP^D^ T1 and T2 are related to human PrP^D^ types 1 and 2, respectively. In contrast, phenotype T3 is the most divergent, especially for spongiform degeneration and PrP^D^ deposition, mostly limited to white matter regions, and electrophoretic profile, where resPrP^D^ recovered as a band of 7 kDa, was the major component shared with the complex pattern of VPSPr resPrP^D^. The T3 histopathologic phenotypes including the PrP immunostaining pattern matched also the bank vole phenotype of GSS^A117V^ and GSS^P102L^ associated with the 8-kDa fragment only (*16*). The exceedingly low representation of the totPrP^D^ and resPrP^D^ components of total PrP in T3 is reminiscent of the corresponding data reported in VPSPr-VV, in which totPrP^D^ accounted for 3.4% and resPrP^D^ for 0.83% of total PrP (*6*). The marked underrepresentation of totPrP^D^ and resPrP^D^ in T3 is especially puzzling considering that attack rate and survival time are not very different from those of T2 and T1, respectively. The apparent relative high efficiency of T3 might be explained by the high representation of oligomers (*36*). Alternatively, the T3 underrepresentation of totPrP^D^ relative to total PrP might reflect the lack of PrP^C^ down-regulation by T3 compared with T1 and T2, which would result in the relative increase of the total PrP pool (*33*,*34*).

A mechanism put forward for the lack of fidelity in cross-species transmission of the prion strain (*25*,*35*–*37*) is based on evidence that the dominant strain is selected from an array of strains that persist as substrains. In cross-species transmissions, substrains may be selected over the dominant strain (*38*–*40*). In the context of VPSPr, this mechanism is particularly intriguing, given that small quantities of PrP^D^ conformers with electrophoretic mobilities similar to those of human PrP^D^ types 1 were originally observed in a few cases by Gambetti et al (*6*); more recently, the presence of PrP^D^ type 2 in VPSPr, mostly in subcortical nuclei and in cerebellum, has been reported (*11*,*12*). These 2 components would be propagated faithfully in T1 and T2, and T3, which consistently shares only the 7-kDa fragment with the VPSPr resPrP^D^, might represent the selective amplification of this GSS-like VPSPr component, perhaps because of the unsuccessful attempt to fully reproduce the dominant strain associated with this disease. We and others have occasionally observed an underrepresented 7-kDa fragment in sCJDMM1 (*41*; S. Notari, P. Gambetti, P. Parchi, unpub. data). Thus, it is tempting to speculate that the 7-kDa fragment observed in bank voles inoculated with sCJDMM1 and sCJDMV1 prions is related to the presence and possibly the infectivity of such fragment in the sCJDMM(MV)1 subtype.

In conclusion, on the basis of the first full transmission of VPSPr, our study confirms the permissiveness of bank voles to human prion diseases and suggests that bank voles are competent to reveal minor strain variants in prion diseases, such as resPrP^D^ types 1 and 2 reported in VPSPr and, possibly, the ≈7-kDa fragment observed in sCJDMM1 and sCJDMV1. However, our study also underscores the limited competence of bank vole PrP^C^ to faithfully reproduce the multiband profile of VPSPr resPrP^D^ that probably reflects the complex conformation of the prion seed in this disease.

Appendix Additional details on variable protease-sensitive prionopathy transmission to bank voles.
