# Supplementary material for: Variable Protease-Sensitive Prionopathy Transmission to Bank Voles
Source: Emerg Infect Dis. 2019 Jan;25(1):73–81. doi: 10.3201/eid2501.180807 (PMC6302590; doi:10.3201/eid2501.180807)
Supplement: Appendix — Additional details on variable protease-sensitive prionopathy transmission to bank voles. [file 18-0807-Techapp-s1.pdf]

Article DOI: https://doi.org/10.3201/eid2501.180807

Variable Protease-Sensitive Prionopathy Transmission to Bank Voles

Appendix

Supplemental Materials and Methods

Tissue samples

Previously Published results of primary transmission of one case with definite sCJDMM1 and one with sCJDMV1 (*1*,*2*) were used as controls. Second passage inocula used for second passage were obtained from prion positive bank voles challenged at first passage. All procedures were performed under protocols approved by the Institutional Review Board of Case Western Reserve University. Written informed consent for research was obtained from all patients or legal guardians according to the declaration of Helsinki. All patients' data and samples were coded and handled in accordance with NIH guidelines to protect patients' identities.

Animals

Bank voles 109M and 109I were obtained from the breeding colony of Istituto Superiore di Sanità (ISS). The research protocol, approved by the Service for Biotechnology and Animal Welfare of the ISS and authorized by the Italian Ministry of Health, adhered to the guidelines contained in the Italian Legislative Decree 116/92, which transposed the European Directive 86/609/EEC on Laboratory Animal Protection, and then in the Legislative Decree 26/2014, which transposed the European Directive 2010/63/UE on Laboratory Animal Protection.

Intracerebral Inoculations

For first passage, 10% brain homogenate in phosphate buffer saline (PBS) was prepared from frontal cortex of VPSPr cases #1–5 or from putamen of VPSPr cases #6 and #7, as well as from frontal cortex of two cases of sCJDMV1 used as control. Similar preparations from half bank vole prion positive brains were used for second passage. Eight-week-old bv109M and bv109I were inoculated with 20 µl brain homogenate into the left cerebral hemisphere, under ketamine anesthesia (ketamine 0.1 µg/g). The animals were examined twice a week until

neurologic signs appeared, and daily thereafter. Diseased animals were culled with carbon dioxide at the terminal stage of the disease, but before neurologic impairment was such as to compromise their welfare, in particular their ability to drink and feed adequately. Survival time was calculated as the interval between inoculation and culling or death. Postmortem, brain was unequally bisected according to a parasagittal plane. The smaller portion was stored at –80°C, the larger fixed in formalin. Positive bank voles were defined as prion positive by Western blot and/or histopathology and immunohistochemical examinations.

Histopathology, immunohistochemistry, lesion profile and PET blot.

Coronal sections were obtained from four antero-posterior levels including: (i) telencephalon at midlevel of caudate nucleus, (ii) diencephalon at midlevel of thalamus, (iii) midbrain and (iv) hindbrain at midlevel of medulla and cerebellum. Microscopic examinations were carried out on sections stained with H.E. or probed with the monoclonal antibody (mAb) to PrP SAF-84 (*1*,*3*). In more detail, sections for immunohistochemistry were deparaffinized, rehydrated and immersed in Tris-buffered-saline Tween 20 and endogenous peroxidase blocked by hydrogen peroxide 8% (Fisher) for 10 minutes. Sections were immersed in 1.5 mmol/L hydrochloric acid, microwaved for 15 minutes and incubated with antibody (Ab) SAF84 at 1: 350 for 1 hour. Sections were then washed, incubated with horseradish peroxidase polymer for 30 minutes (Envision Flex, Dako) and treated with 3′-diaminobenzidine (Envision Flex, Dako) to show the immunostaining.

Lesion profiles were constructed performing semi-quantitative evaluation of spongiform degeneration by comparing 5 hematoxylin and eosin-stained sections as previously described (*4*). Briefly, 9 areas of gray matter and 1 of white matter were examined and separate score was given for the severity of spongiform degeneration.

Additional lesion profiles were produced at the ISS by following the procedure for bank voles previously reported (*5*).

PET blot was carried out on coronal sections, as previously described (*5*). Briefly, sections were collected on prewetted 0.45-lm-pore nitrocellulose membranes (Schleicher & Schuell, Dassel, Germany). Membranes were dried for 24 h at 55°C. Membrane treatments, proteinase K digestion (50 lg/ml) and immunodetection with NBT/BCIP were performed as previously described. Monoclonal mAb 6C2 was used as primary antibody.

Western blot

Brain homogenates (10%, w/v) were prepared on ice in lysis buffer with high buffer capacity (LB 100) (100 mM Tris, 100 mM NaCl, 10 mM EDTA, 0.5% Nonidet P-40, 0.5% sodium deoxycholate), pH 6.9. Aliquots were treated for 1 h at 37°C with PK (Roche Diagnostics) at the concentration of 500 μg/ml. Protease digestion was terminated by the addition of 2 mM phenylmethylsulfonyl fluoride (PMSF). Samples were diluted in sample buffer (final concentration: 3% SDS, 4% β-mercaptoethanol, 10% glycerol, 2 mM EDTA, 62.5 mM Tris, pH 6.8) and boiled for 8 min before loading. For protein N-deglycosylation, samples were denatured and incubated in the presence of recombinant peptide N glycosidase F (PNGase F) according to the manufacturer's protocol (New England Biolabs). Protein samples were separated with Tris-Glycine SDS-PAGE in 15% Criterion Tris-HCl polyacrylamide precast gels (Bio-Rad Laboratories, Hercules, CA, USA) and transferred to Immobilon-P PVDF transfer membrane (EMD-Millipore, Billerica, MA, USA) for 2 h at 60 V, blocked with 5% nonfat dry milk in 0.1% Tween 20-Tris-buffered saline, pH 7.5, and probed with the appropriate Ab. The immunoreactivity was visualized by enhanced chemiluminescence (Pierce ECL 2, Fisher Scientific, Hampton, NH, USA) on Kodak BioMax Light films (Eastman Kodak Co., Rochester, NY, USA).

Preparation of insoluble fraction

Brain homogenates (10% w/v) in LB100 pH 6.9 were incubated, with frequently mixing, for 20 min at 4° and centrifuged at 1,000 x g for 5 min at 4°C. The obtained supernatant, identified as S1, was ultracentrifuged at 100,000 x g for 1 h at 4°C to generate a pellet (P2) for each sample, representing the insoluble fraction of total PrP, i.e., total PrP^D ($totPrP^D$) and a small amount of PrPC, that remains insoluble in our procedure. The P2 was resuspended in sample buffer, boiled and analyzed by Western blot. Densitometry was performed on the scanned film images by UN-SCAN-IT software (Silk Scientific). The average insoluble PrP^C measured from the uninoculated mice was then subtracted from the total insoluble PrP of each group to obtain the specific $totPrP^D$.

Monoclonal Antibodies

Ab to bank vole PrP 9A2 (to epitopes 99–101) (Vageningen University and Research, Lelystad, Netherland), 12B2 (to epitopes 89–93) (Vageningen University and Research, Lelystad, Netherland) and SAF84 (163–169) (Cayman Chem. Ann Arbor, Michigan) were used

for tissue immunostaining and Western blot. Ab 1E4 (to epitopes 97–108) (Cell Sciences, Inc., Canton, MA, USA) was used to probe Western blot of VPSPr used as control, and Ab 6C2 (to epitopes 111–116) (Vageningen University and Research, Lelystad, Netherland) to probe PET blot.

References

1. Nonno R, Angelo Di Bari M, Agrimi U, Pirisinu L. Transmissibility of Gerstmann-Sträussler-Scheinker syndrome in rodent models: new insights into the molecular underpinnings of prion infectivity. Prion. 2016;10:421–33. PubMed http://dx.doi.org/10.1080/19336896.2016.1239686

2. Rossi M, Saverioni D, Di Bari M, Baiardi S, Lemstra AW, Pirisinu L, et al. Atypical Creutzfeldt-Jakob disease with PrP-amyloid plaques in white matter: molecular characterization and transmission to bank voles show the M1 strain signature. Acta Neuropathol Commun. 2017;5:87. PubMed http://dx.doi.org/10.1186/s40478-017-0496-7

3. Pastore M, Chin SS, Bell KL, Dong Z, Yang Q, Yang L, et al. Creutzfeldt-Jakob disease (CJD) with a mutation at codon 148 of prion protein gene: relationship with sporadic CJD. Am J Pathol. 2005;167:1729–38. PubMed http://dx.doi.org/10.1016/S0002-9440(10)61254-0

4. Kim JI, Cali I, Surewicz K, Kong Q, Raymond GJ, Atarashi R, et al. Mammalian prions generated from bacterially expressed prion protein in the absence of any mammalian cofactors. J Biol Chem. 2010;285:14083–7. PubMed http://dx.doi.org/10.1074/jbc.C110.113464

5. Di Bari MA, Nonno R, Agrimi U. The mouse model for scrapie: inoculation, clinical scoring, and histopathological techniques. Methods Mol. Biol. 2012;849:453–71. http://dx.doi.org/10.1007/978-1-61779-551-0_31 PMID: 22528109

6. Nonno R, Di Bari MA, Cardone F, Vaccari G, Fazzi P, Dell'Omo G, et al. Efficient transmission and characterization of Creutzfeldt-Jakob disease strains in bank voles. PLoS Pathog. 2006;2:e12. PubMed http://dx.doi.org/10.1371/journal.ppat.0020012

Appendix Table 1. Simplified classification of sporadic human prion diseases

Type	Subtype*
Sporadic Creutzfeldt-Jakob disease (sCJD)	sCJDMM(MV)1[1,2]; sCJDMM2; sCJFMV2; sCJDVV1; sCJDVV2
Sporadic fatal insomnia (sFI)	sFI(MM2)
Variably protease sensitive prionopathy (VPSPr)	VPSPrMM; VPSPrMV; VPSPrVV

*MM, MV and VV refer to the PrP 129 genotype; 1 and 2 refer to types or strain of the associated protease-resistant PrP. 2 sCJDMM1 and sCJDMV1 are combined as sCJDMM(MV)1 because they share the phenotype.

Appendix Table 2. Synopsis of data from all VPSPr inoculations to bank voles

			Bv109I				Bv109M			
Inoculum	Genotype	Passage	Attack rate	T1	T2	T3	Attack rate	T1	T2	T3
#1	M129M	*1° pass.*	1/10	839	839		1/8	356		
		2° pass.	nd	nd			11/11	148±12		
#2	M129M	*1° pass.*	6/10	901		413±102	0/7			
		2° pass.	9/9	nd		247±35	nd			
#3	M129V	*1° pass.*	14/25	458±137	839		2/8	290,588		
		2° pass.	10/10	195±9	nd		11/11	142±11		
#4	M129V	*1° pass.*	3/9		826±91		0/11			
		2° pass.	5/5		215±11		nd			
#5	M129V	*1° pass.*	3/10		855,1061	554	0/11			
		2° pass.	9/9		407±36*	nd	nd			
#6	V129V	*1° pass.*	1/8			535	0/9			
		2° pass.	nd			nd	nd			
#7	V129V	*1° pass.*	1/10			596	0/5			
		2° pass.	9/9			255±24	nd			

*Secondary passage was performed using the brain of vole with 855 d.p.i. that showed low PrPSc amount. This might explain the longer survival time observed compared to VPsPr #4 in Bv109I.

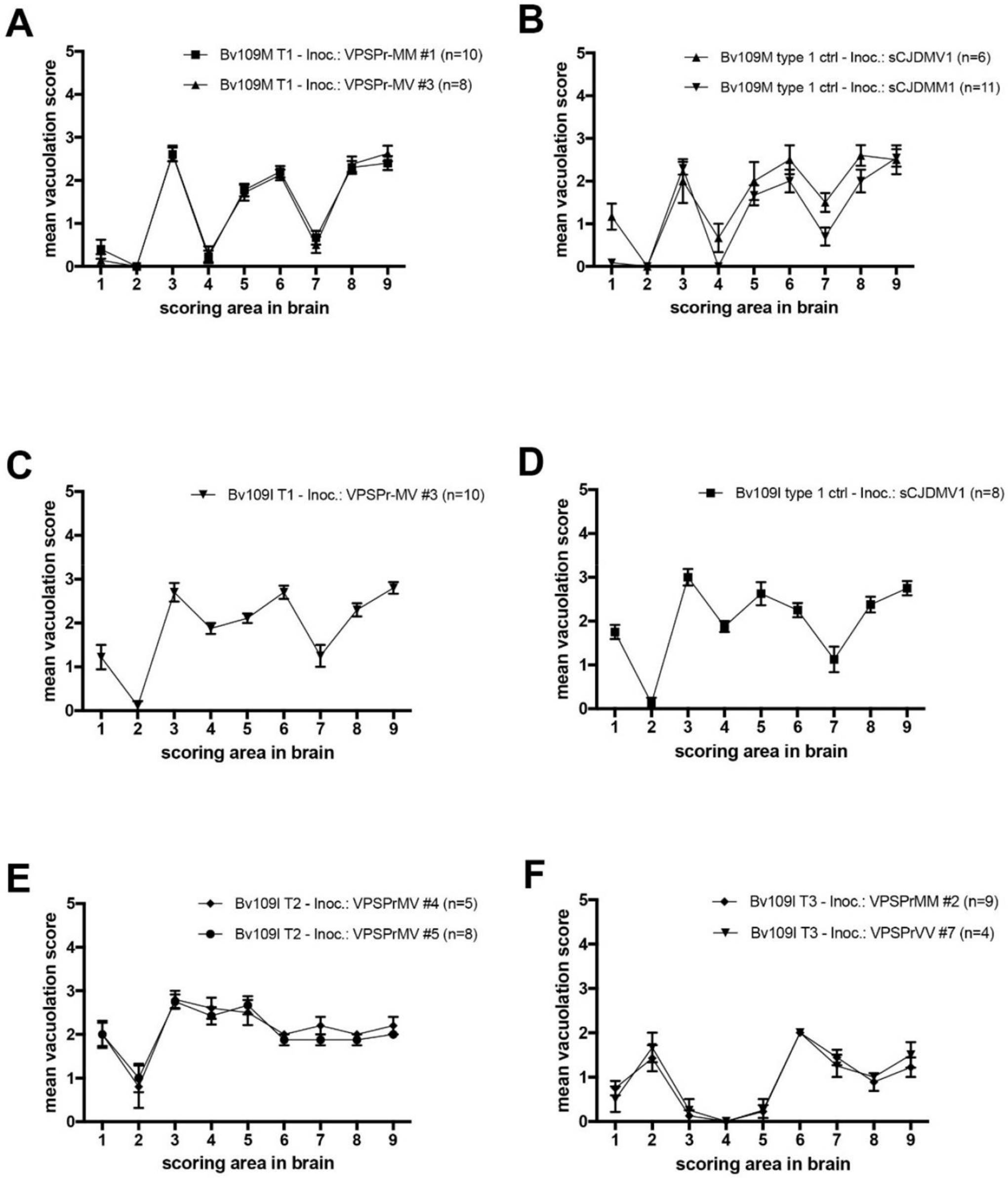

Appendix Figure 1. Alternative lesion profiles of VPSPr and type controls in bv109M and bv109I constructed according to procedures previously used with bank voles. A and B) Profiles of second passage in bv109M of T1 from VPSPrMM and VPSPrMV compared with matching profiles from sCJDMM1 and sCJDMV1 (*6*). C and D) As in A and B above in bv109I of T1 from VPSPr-MV compared with the profile from sCJDMV1 (*2*). E and F) Second passage in bv109I of T2 of two cases of VPSPrMV (E) and of T3 (F) from VPSPrMM and VPSPrVV. Brain-scoring areas: medulla (1), cerebellum (2), superior colliculus (3), hypothalamus (4), thalamus (5), hippocampus (6), septum (7), retrosplenial and adjacent motor cortex (8), cingulate and adjacent motor cortex (9).

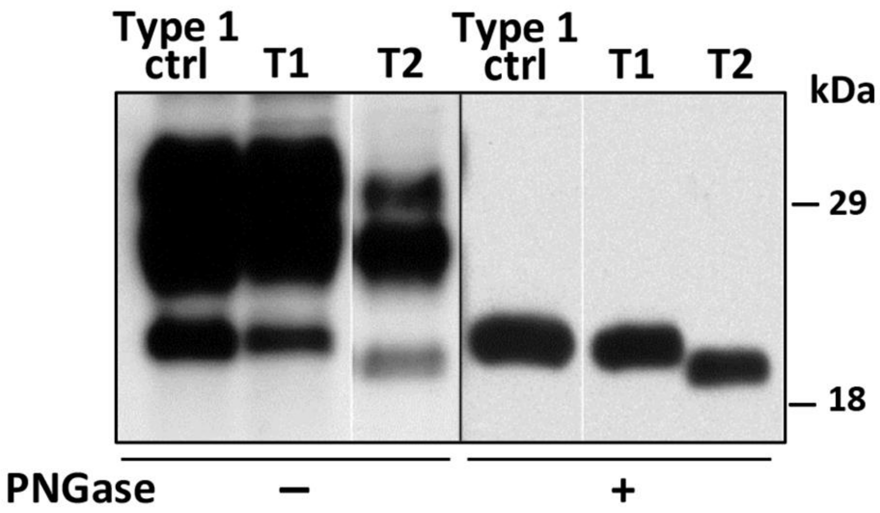

Appendix Figure 2. Deglycosylation of resPrP^D associated with T1 and T2 phenotypes**.** Following digestion of PK-treated BH with PNGase F, the 3 bands populated by the 3 resPrP^D glycoforms of T1 and T2 were reduced to single 21 kDa and 19 kD, respectively, matching the unglycosylated isoform. The Type 1 ctrl (from bv109I inoculated with sCJDMV1), treated and untreated with PNGase F, is shown as control. Ab 9A2.

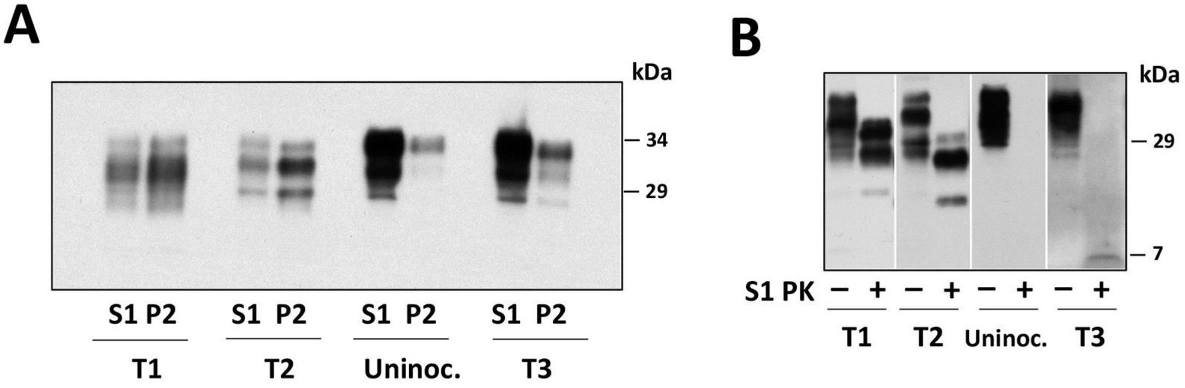

Appendix Figure 3. Immunoblots of PrP individual components separated from T1-T3 and uninoculated control preparations used in Figure 6. A) TotPrP (S1, comprising protease-sensitive and –resistant PrP^D along with PrP^C) and insoluble PrP (P2, comprising protease-sensitive and –resistant PrP^D and a small amount of PrP^C insoluble in our procudure); B) TotPrP PK-untreated (S1 PK-) and PK-treated (S1 PK+, harboring exclusively PK-resistant PrP^D). TotPrP samples (S1 and S1 PK-) were loaded at a dilution 1:2 of the respective P2 and S1 PK+ samples. Blots (T1-T3 and Uninoc.) shown in B underwent different exposures. Note the distinct profiles of totPrP^D (P2) in T1-T3 and that the pronounced divergence in glycoform ratio between T1 and T2 clearly emerges only after PK treatment (compare P2 in A with S1 PK+ in B).
